# Sexual dysfunction in a sample of Egyptian patients with Parkinson’s disease

**DOI:** 10.1007/s10072-023-07091-2

**Published:** 2023-10-02

**Authors:** Heba Assem Deraz Abdelhalim Deraz, Hanan Abdalla Hassan Amer, Muhammad Ramadan Suleiman, Ahmed Dahshan

**Affiliations:** Cairo University, Giza, Egypt

**Keywords:** Parkinson disease, Sexual dysfunction, Disease severity, Arabic Female Sexual Function Index, International Index of Erectile Function

## Abstract

**Background:**

Sexual dysfunction (SD) is a common, yet underdiagnosed problem in Parkinson Disease (PD) patients. It can negatively impact their quality of life (QoL) and clinical outcome. we tried to assess SD in a group of Egyptian PD patients.

**Methods:**

The study is a case–control, cross-sectional study that included 200 participants, consisting of 100 PD patients and 100 matched healthy controls. Social, demographic information, and clinical variables were collected from both groups. Sexual functions were assessed using the Arabic Female Sexual Function Index (ArFSFI), and the Arabic version of International Index of Erectile Function (IIEF).

**Results:**

Women with PD scored worse on FSFI total score compared to controls (*p* < 0.001). Regarding the FSFI domains, they scored significantly lower in individual domains of desire (*p* < 0.001), arousal (*p* < 0.001), lubrication (*p* = 0.006), orgasm (*p* < 0.001), satisfaction (*p* < 0.001), and pain (*p* = 0.003), compared with controls. Men with PD scored worse on IIEF total scores compared to controls (*p* < 0.001). They showed significantly worse scores of erectile functions (*p* < 0.001), orgasmic function (*p* < 0.001), sexual desire (*p* < 0.001), intercourse satisfaction (*p* < 0.001), and overall satisfaction (*p* < 0.001). Both groups reported significant effect of SD on their QoL. There was a significant correlation between disease severity and SD.

**Conclusion:**

SD is common in PD patients. It negatively impacts their QoL and partnership. Healthcare professionals should initiate conversations about SD with the patients and provide appropriate education and treatment options.

## Introduction

Parkinson's disease (PD) is a chronic neurodegenerative disorder characterized by the cardinal motor symptoms of bradykinesia, rigidity, tremor, and postural instability. With a prevalence of 100–300 per 100,000, PD is the second most common neurodegenerative disorder after Alzheimer's disease [1]. As life expectancy continues to increase, it is projected that the number of individuals with PD will double from approximately 6 million to 12 million by 2040 [2]. Although motor symptoms are the hallmark of PD, non-motor symptoms (NMS) such as sleep disorders, affective disorders, gastrointestinal symptoms, and sexual dysfunction (SD) are common and can have a significant impact on quality of life (QoL) [3]. SD is particularly prevalent in PD, with up to 79% of men and 87% of women affected. Loss of libido is the most commonly reported SD in women, with a prevalence twice as high as that of the general population, and erectile dysfunction is the most common SD in men with PD. SD can have a negative impact on the QoL and relationships of people with PD [4]. However, despite its high prevalence and impact, SD is often underreported and/or underrecognized in clinical practice and is not openly addressed by patients or physicians [5]. In particular, the topic of sexuality in PD is still under investigated in Egypt with scarcity of research on this area, and only a limited number of studies have addressed this topic so far [6, 7]. More research is needed to address this issue to allow a better understanding of the sexual problems and their impact in PD in the Egyptian population [8]. In this study, we aimed to investigate the prevalence of SD in a sample of Egyptian patients with PD and to explore the relationship between the pattern of SD and disease characteristics. By addressing this important yet underrecognized issue, we hope to improve the QoL of individuals with PD and enhance their overall management.

## Methods

The study is an observational, case–control, cross-sectional study that included 200 participants, consisting of 100 Parkinson's disease (PD) patients during their regular visits to the movement disorder clinic to the Neurology department at Kasr Al-Ainy School of Medicine, Cairo University Hospitals and 100 matched healthy volunteers as controls. The patients were diagnosed with PD based on the UK Parkinson’s Disease Society Brain Bank diagnostic criteria [9]. The study population was divided into two groups, the Patients Group (50 male and 50 female PD patients) and the Control Group (50 male and 50 female healthy volunteers). The study included individuals aged between 40–80 years of both sexes. Participants with major medical disorders uncontrolled by current pharmacological and non-pharmacological regimens (e.g. uncontrolled diabetes mellitus, hepatic or renal impairment) were excluded. Patients with cognitive deterioration precluding comprehension of the study scope and its instruments and those with severe motor impairment (Hoehn and Yahr (HY) stage > 4) were also excluded from our study. Social and demographic information from both groups, as well as clinical variables, were collected which included age of onset, disease duration, disease subtype, current antiparkinsonian medication, total Unified Parkinson's Disease Rating Scale (UPDRS) score [10], and Hoehn and Yahr (HY) stage [11]. Comorbidities, other current medication, and menopausal status were also obtained from patients and controls, patients were in the on state during the visit. And a written consent was obtained from all participants.

Sexual functions of PD patients and controls were assessed using two instruments: the Arabic Female Sexual Function Index (ArFSFI) [12], and the Arabic version of International Index of Erectile Function (IIEF) [13]. The ArFSFI is a brief multidimensional self-report questionnaire that assesses six domains of female sexual function, while the IIEF consists of 15 items investigating five domains of male sexual function. Both of the IIEF and FSFI scales only measure sexual function in these domains over the previous 4 weeks. Patients were asked to determine the effect of their current sexual problems on their overall quality of life (QoL) and partnership. A scale from 0 to 10, developed by the author, was used, where 0 represented ‘not affecting’ and 10 represented ‘very affecting’. Data were coded and entered using the statistical package for the Social Sciences (SPSS) version 28 (IBM Corp., Armonk, NY, USA). Data was summarized using mean and standard deviation for quantitative variables and frequencies (number of cases) and relative frequencies (percentages) for categorical variables. Comparisons between groups were done using unpaired t test when comparing 2 groups and analysis of variance (ANOVA) with multiple comparisons post hoc test when comparing more than 2 groups [14]. For comparing categorical data, Chi square (χ2) test was performed. Exact test was used instead when the expected frequency is less than 5 [15]. Correlations between quantitative variables were done using Pearson correlation coefficient [16]. *P*-values less than 0.05 were considered statistically significant.

## Results

### Demographic characteristics of study subjects

Table 1 shows summary of demographic characteristics of the study subjects. Patients did not differ statistically from controls with respect to education levels, employment status, marital status, and residency.

**Table 1:** Demographic characteristics of study subjects

	Female Patients (*n* = 50)	Female Controls (*n* = 50)	*P* value	Male patients (*n* = 50)	Male Controls (*n* = 50)	*P* value
Age (years), mean ± SD	60.10 ± 6.78	58.94 ± 7.18	0.408	67.82 ± 4.48	68.66 ± 5.16	0.387
Education, %						
Illiterate	44.0	40.0	0.935	10.0	16.0	0.645
Elementary	24.0	22.0		38.0	38.0	
High school, Bachelor	28.0	32.0		52.0	46.0	
Postgraduate degree	4.0	6.0		0.0	0.0	
Employment, %						
Unemployed	62.0	56.0	0.345	8.0	12.0	0.356
Employed	22.0	34.0		68.0	54.0	
Retired	16.0	10.0		24.0	34.0	
Residency, %						
City (urban)	70.0	74.0	0.656	50.0	46.0	0.689
Village (rural)	30.0	26.0		50.0	54.0	
Marital status, %						
Single	0.0	2.0	1	0.0	0.0	0.349
Married	92.0	94.0		80.0	72.0	
Divorced	2.0	0.0		0.0	0.0	
Widowed	6.0	4.0		20.0	28.0	
Menopausal females, %	96.0	96.0	1	-	-	-
Concurrent medications, %						
None	46.0	54.0	0.304	42.0	40.0	0.975
Antidepressants	16.0	6.0		8.0	6.0	
Antipsychotics	4.0	0.0		0.0	0.0	
Others	34.0	40.0		50.0	54.0	
Comorbidities, %						
None	64.0	64.0	1	52.0	48.0	0.988
Hypertension	24.0	24.0		28.8	28.0	
Diabetes mellitus	6.0	8.0		12.0	14.0	
Kidney disease	2.0	0.0		8.0	8.0	
Liver disease	2.0	2.0		0.0	0.0	
Cardiac disease	0.0	2.0		0.0	0.0	

### Clinical characteristics of patients’ group

In women with PD, the mean age of disease onset was 55.76 years (SD: 7.10; range: 38–68) and the mean disease duration was 4.34 years (SD: 2.79; range: 1–13), while in male patients, the mean age of PD onset was 63.32 (SD: 4.87; range: 55–71) and the mean disease duration was 4.50 (SD: 2.06; range: 1–10). Twenty-four (48%) women with PD presented with a tremor-predominant disease, 16 (32%) with akinetic-rigid syndrome, and 10 (20%) with a mixed type of disease. Regarding men with PD, 19 (38%) presented with akinetic-rigid syndrome, 16 (32%) with a tremoric disease, and 15 (30%) with a mixed disease subtype. Concerning disease severity, the mean "on" total UPDRS score was 47.66 ± 15.66 in women with PD, and the mean HY stage was 2.57 ± 0.59. Meanwhile, male patients showed a mean "on" total UPDRS score of 94.82 ± 15.81 and a mean HY stage of 2.20 ± 0.83. Forty-eight women (96%) and 46 men (92%) with PD were currently taking dopaminergic medications. Table 2 shows summary of clinical characteristics of patients’ group.

**Table 2:** Clinical characteristics of patients’ group

	Female patients (*n* = 50)	Male patients (*n* = 50)
Age of PD onset (years), mean ± SD	55.76 ± 7.10	63.32 ± 4.87
Disease duration (years), mean ± SD	4.34 ± 2.79	4.50 ± 2.06
PD subtype, %		
Akinetic-rigid	32.0	38.0
Tremor-dominant	48.0	32.0
Mixed	20.0	30.0
*On* total UPDRS score, mean ± SD	47.66 ± 15.66	94.82 ± 15.81
HY stage, mean ± SD	2.57 ± 0.59	2.20 ± 0.83
Dopaminergic drugs, %	96	92

### Assessment of sexual dysfunction

All patients and control subjects completed the sexual function assessment scales (FSFI in women and IIEF in men). Women with PD scored worse on FSFI total score compared to controls (*p* < 0.001). The mean FSFI total score in patients was 16.83 ± 6.43, while that in control subjects was 22.11 ± 6.49. Regarding the FSFI domains, women with PD scored significantly lower in individual domains of desire (*p* < 0.001), arousal (*p* < 0.001), lubrication (*p* = 0.006), orgasm (*p* < 0.001), satisfaction (*p* < 0.001), and pain (*p* = 0.003), compared with controls. Similarly, men with PD scored worse on IIEF total scores compared to controls (*p* < 0.001). The mean IIEF total score in male patients was 13.74 ± 3.76, while in healthy men, the total score was 18.44 ± 2.71. Male patients with PD showed significantly worse scores of erectile functions (*p* < 0.001), orgasmic function (*p* < 0.001), sexual desire (*p* < 0.001), intercourse satisfaction (*p* < 0.001), and overall satisfaction (*p* < 0.001), compared to healthy male controls. Patients were asked to determine the effect of their current sexual problems on their overall quality of life (QoL) and partnership. In women with PD, the mean effect of SD on the overall QoL was 3.94 ± 2.07 and the mean effect of SD on partnership was 4.40 ± 2.48. Regarding men with PD, the mean effect of SD overall QoL was 5.28 ± 2.15 and the mean effect of SD on partnership was 4.64 ± 2.48. In women with PD, the mean duration of SD was 4.60 years (SD: 2.20; range: 1–10), while in male patients, the mean duration of SD was 3.06 years (SD: 1.63; range: 1–8). Fifteen men with PD (30.0%) sought medical advice regarding their current sexual problems, and all of those who sought consultations (30.0%) received medical treatment for their sexual problems. However, only one woman with PD (2.0%) sought medical consultation for her sexual problems, and none of the female patients received any specific treatment for SD. Most women with PD related their current SD to aging (46.0%), 18 patients (36.0%) related their sexual problems to PD, 4 patients (8.0%) related SD to their current antiparkinsonian medications, and 10 patients (10.0%) related SD to other causes. Similarly, most men with PD related their sexual problems to normal aging (44.0%). Sixteen men with PD (32.0%) related SD to PD, 5 patients (10.0%) related SD to antiparkinsonian medications, and 7 patients (14.0%) related sexual problems to other causes. Table 3 shows overall sexual function of patients with PD.

**Table 3:** Sexual functions of patients

	Female Patients (*n* = 50)		Male patients (*n* = 50)
FSFI total score, mean ± SD	16.83 ± 6.43	IIEF total score, mean ± SD	13.74 ± 3.76
Duration of SD, mean ± SD	4.60 ± 2.20	Duration of SD, mean SD	3.06 ± 1.63
Effect of SD on QoL, mean ± SD	3.94 ± 2.07	Effect of SD on QoL, mean ± SD	5.28 ± 2.15
Effect of SD on partnership, mean ± SD	4.40 ± 2.48	Effect of SD on partnership, mean ± SD	4.64 ± 2.48
SD related to, %		SD related to, %	
Aging	46.0	Aging	44.0
PD	36.0	PD	32.0
Antiparkinsonian drugs	8.0	Antiparkinsonian drugs	10.0
Other causes	10.0	Other causes	14.02

### Correlations

**Fig. 1:**
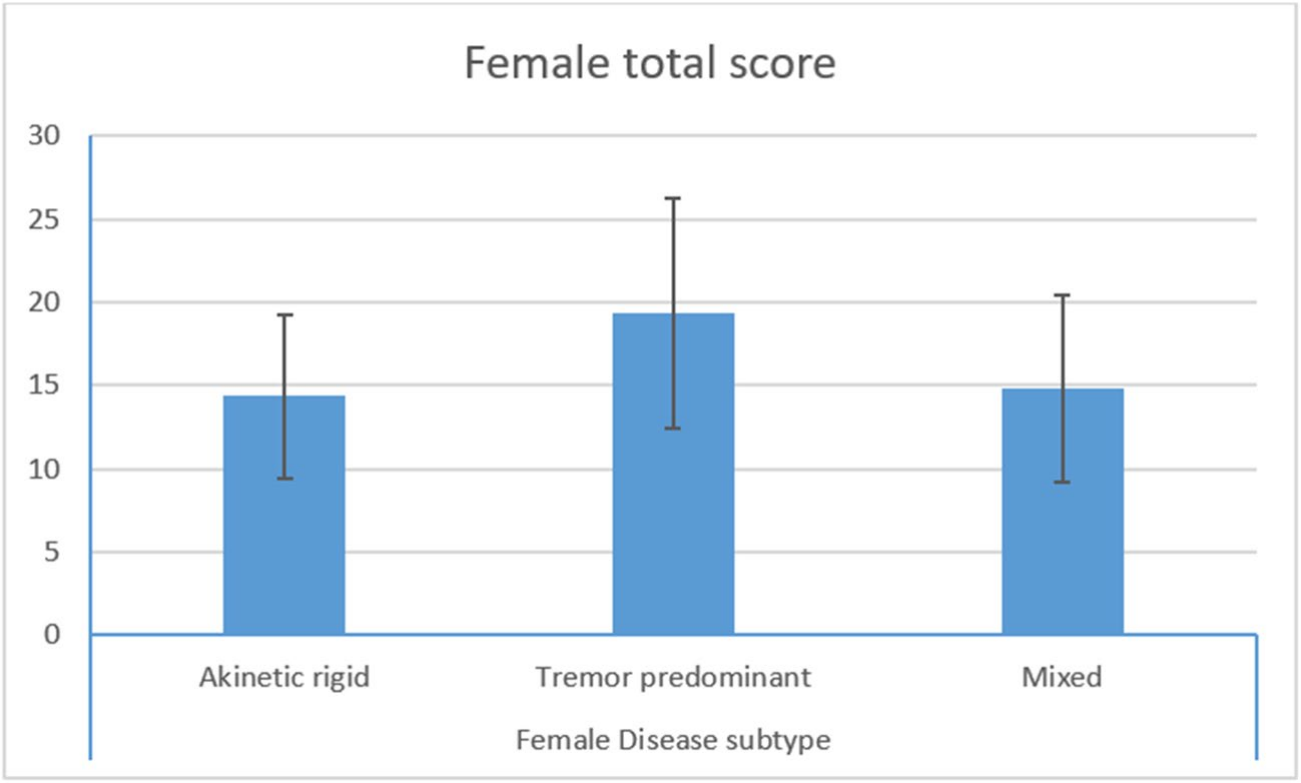
Post hoc comparison between PD subtypes regarding mean FSFI total scores

We assessed the correlation between the scores of SD scales and PD severity, as measured by "on" total UPDRS score and HY stage. In women with PD, a strong negative correlation was found between the FSFI total score and both the total UPDRS score (r = -0.347, *p* = 0.014) and HY stage (r = -0.294, *p* = 0.038). Men with PD also showed a statistically significant negative correlation between the IIEF total scores and HY stage (r =—0.389, *p* = 0.005). There was no statistical correlation between IIEF score and total UPDRS score (r =—0.267, *p* = 0.061). The IIEF total score was negatively correlated with PD duration (r = -0.359, *p* = 0.010). However, the FSFI scores showed no significant correlation with disease duration. Furthermore, we analyzed the relation between PD subtypes and the SD scales total scores. A significant difference in the mean FSFI total scores (*p* = 0.027) was found between different disease subtypes in female patients. Post hoc pair wise comparisons revealed that both the akinetic-rigid syndrome and mixed PD subtype were associated with worse FSFI total scores compared to the tremor-dominant disease (*p* = 0.043, 0.043 respectively) (Fig. 1).

No differences in IIEF total scores were found between PD subtypes in male patients. IIEF Scores of Male PD Subgroups:

- Akinetic-Rigid Syndrome: Mean IIEF score 12.74 ± 2.64
- Tremor-Dominant Disease: Mean IIEF score 13.13 ± 4.05
- Mixed Disease Subtype: Mean IIEF score 13.67 ± 3.13

There is no statistical significance when comparing these groups.

## Discussion

Sexual dysfunction (SD) is a common, yet under-reported, non-motor symptom (NMS) of Parkinson’s disease (PD). Addressing such a problem and asking about it should be a constant part during assessment and follow up of parkinsonian patients. It is usually underestimated due to either patients’ special issues as social issues or physician neglection of the problem in comparison to other motor and non-motor complaints. This problem could have a severe impact on quality of life of patients and could have a serious impact on other manifestations which add to patients suffering. Several mechanisms of sexual dysfunction in PD were reported, independent of disease severity, including autonomic dysfunction, central dopaminergic deficiency, psychosocial factors, and medication side effects [17]. In the present study, we found that sexual dysfunction is prevalent among both male and female patients with PD. Our findings are consistent with previous work that also reviled the high prevalence of SD in patients with PD and the effect of PD on sexual functions [18–21]. Our results also showed that patients with PD have significantly worse sexual function compared to healthy controls, with lower scores in desire, arousal, lubrication, orgasm, satisfaction, and pain in women, and lower scores in erectile function, orgasmic function, sexual desire, intercourse satisfaction, and overall satisfaction in men, which goes with previous studies that addressed sexual domains most affected by PD [4, 18, 22, 23]. Sexual dysfunctions have a high impact on quality of life (QoL), which has been previously addressed in patients with PD [4, 5]. In our sample, most of the patients declared that their sexual problems negatively affect their relationship with the partner and overall QoL. Reduced sexual activity and satisfaction in patients with PD could be related to physical disability related to the disease, as well to other PD non-motor symptoms as depression, lack of motivation, and sleep disturbances which may lead to bed separation [24]. We also found that most patients with PD attribute their sexual dysfunction to aging rather than to the disease or its treatment, which highlights the importance of patient education on the multiple factors that could be related to sexual dysfunction in PD. Aging has been proved to be a main factor regarding sexual dysfunction as shown by other studies [6, 25]. Sakakibara and colleagues in 2001, compared the sexual functions in different age groups (30 s, 40 s, 50 s, and 60 s) and reported significant decline in the frequency of sexual intercourse and orgasm in elder PD patients [26]. Another study demonstrated more frequent and severe sexual dysfunction in late onset PD than early onset PD patients and attributed differences to the age of onset [27]. In the same context, we have to underline that sexuality is still an embarrassing topic for women. Only 2% of women sought medical advice regarding their sexual problems, compared to 30% of men in our sample. This issue should encourage medical providers to discuss symptoms of sexual dysfunction with their patients with PD. This may be challenging since this topic is not covered in the UPDRS, in addition to cultural barriers rendering patients, particularly women, in addressing their sexual problems and habits. However, going through a routine symptom review during regular visits and referring patients to sex therapy, couple therapy, and behavioral therapy, where appropriate, may be helpful. Overall, medical providers should be encouraged to initiate conversations about sexual dysfunction with their patients and educate them on the potential causes and treatments available. In our study, sexual functions correlated negatively with disease severity, as assessed by UPDRS and HY stage. Our results suggest that increased severity of motor impairment and decreased overall activities of daily living negatively affect sexual function which seem logical, and these results go side to side with previous studies that reported a correlation between disease severity and sexual dysfunction in patients with PD [7, 28, 29]. These observations may address the importance of motor rehabilitation in improving patient’s sexuality. Furthermore, we reported worse sexual functions with longer disease duration in men with PD; however, no similar correlation was found in female patients. Some studies also showed no correlation between sexual dysfunction and PD duration [18, 30]. This association between disease severity and the occurrence of non-motor symptoms, including autonomic dysfunction, has been widely reported in the literature [31]. As PD progresses, patients often experience a greater burden of non-motor symptoms, including sexual dysfunction, which can significantly impact their quality of life [32]. Kinateder and colleagues found that male patients with ED had a significantly longer PD duration [5]. Although several studies have suggested that the burden of non-motor symptoms as a whole is higher in the akinetic-rigid phenotype compared to tremor-dominant phenotype [33–35], to the best of our knowledge, this is the first study that directly assesses the relationship between PD subtype and sexual dysfunctions. We found that in women with PD, sexual dysfunctions are more prevalent in the akinetic-rigid and mixed subtypes compared to tremor-dominant phenotype. However, no similar correlation was found in men with PD. The observation of differences in sexual dysfunction scores between PD subgroups aligns with our hypothesis that certain motor phenotypes of PD might correlate with distinct patterns of sexual dysfunction. However, the specific reasons behind these differences warrant further investigation. Potential Explanations include neuroanatomical variations which may influence the neural pathways responsible for sexual function, leading to differences in sexual dysfunction [36]. It is also possible that patients with certain PD subtypes receive different medications or experience varying responses to dopaminergic therapy, which could impact sexual function differently [31]. Non-motor symptoms play a crucial role in PD and can significantly affect sexual function. It's plausible that patients with specific PD subtypes may experience more pronounced non-motor symptoms, which could contribute to differences in sexual dysfunction [32]. The identification of these differences in SD scores among PD subgroups underscores the importance of considering PD heterogeneity when assessing sexual dysfunction. Future research should delve deeper into the underlying mechanisms behind these differences, exploring neurobiological, pharmacological, and psychosocial factors.

## Limitations

Our study has some limitations, including a relatively small sample size and a cross-sectional design, which precludes us from drawing causal inferences. Additionally, we only used self-report measures to assess sexual function, which may be subject to recall bias and social desirability bias. The IIEF and FSFI scales used in our study only evaluate subjects with a current partner and with regular intercourse in the 4 weeks before the interview, providing a superficial characterization of non-sexually active patients. However, these limitations are partly overcome by the presence of a control group with matched geographic and sociodemographic characteristics, which makes the assessment of sexual dysfunction in our patients more precise and accurate. Future studies should use objective measures of sexual function and larger sample sizes to confirm our findings.

## Conclusion

Our study highlights the high prevalence of sexual dysfunction in patients with PD and the negative impact it has on their quality of life and partnership. Healthcare professionals should initiate conversations about sexual dysfunction with their patients with PD and provide appropriate education and treatment options.
